# Reproductive Disorders in Domestic Ruminants: A One Health Concern

**DOI:** 10.3390/pathogens11101139

**Published:** 2022-10-01

**Authors:** Valentina Virginia Ebani

**Affiliations:** 1 Department of Veterinary Sciences, University of Pisa, Viale delle Piagge 2, 56124 Pisa, Italy; valentina.virginia.ebani@unipi.it; 2 Centre for Climate Change Impact, University of Pisa, Via del Borghetto 80, 56124 Pisa, Italy

Farm ruminants (cattle, sheep and goats) are an important economic and food source for humans, both in developing countries and in areas with advanced animal husbandry. Reproductive failures in these animals, represented by abortion, stillbirth, premature birth, and infertility, may be related to several issues. Inadequate management of livestock with nutritional deficiencies, and stresses caused by several factors (transport, high or low temperature, high population density), can contribute to reproductive problems in domestic ruminants. However, abortion and other genital disorders are often caused by a plethora of infectious agents that in most cases are also able to infect humans [1]. In this view, reproductive failures are not only a relevant economic issue [2], but also a severe One Health concern.

The primary abortive zoonotic pathogens are *Coxiella burnetii*, *Chlamydia abortus, Listeria monocytogenes, Brucella* spp., *Leptospira* spp., *Salmonella enterica*, *Campylobacter* spp., and *Yersinia enterocolitica*.

These agents affect animal productivity and cause considerable direct and indirect economic losses to farmers, because of the reduced number of births, decreased milk production, and cost due to diagnosis and treatments.

Huge amounts of pathogens are shed by infected female ruminants mainly through abortion and lochiations, but milk, urine, and feces also can contain the bacteria, and thus the risk of infection for farmers and other persons in contact with the animals is very high. Moreover, most of these bacterial pathogens could pass to other animals, such as dogs circulating in farms, which can become an additional source of infection for their owners.

In the literature, several cases of human abortion related to abortive agents of ruminants are reported, and not only in geographic areas where measures of prophylaxis and environmental hygiene are not carefully performed. 

*Coxiella burnetii* has been often associated with adverse pregnancy outcomes worldwide in the cases of women with rural housing, contact with cattle or sheep, cohabitation with pets, and in others without apparent animal contact [3,4]. This pathogen is massively emitted at abortion and during birth in ruminant livestock, but also during the shearing and manipulating of wool and leather carrying contaminated dust and feces of infected ticks [5]. Miscarriage, stillbirth, pre-term delivery, low infant birth weight, and fetal death or malformations (omphalocele, hypospadias, Potter syndrome, congenital hydronephrosis, and syndactyly) and growth retardation have been related to human *C. burnetii* infection [4]. Moreover, C. burnetii in pregnant women increases the risk of maternal chronic Q fever endocarditis [6].

Abortion in women caused by *C. abortus* has been reported, such as a recent case in France in the wife of a goat farmer, who referred to an increased number of abortions in his herd during the previous 2 years and the fact that the goats were not vaccinated against C. abortus [7]. *Chlamydia abortus* infection in pregnant women initially causes influenza-like illness and successively abortion or stillbirth, gestational sepsis, and pelvic inflammatory disease [8]. 

About one-third of reported human listeriosis cases happen during pregnancy, which may result in spontaneous abortion in the second or third trimester [9]. The placenta of a pregnant woman provides a protective environment for the growth of *L. monocytogenes*, thereby resulting in spontaneous abortions, stillbirth, and neonatal infection. The fetus suffers more damage than the pregnant woman with consequent infant mortality [10]. In fact, *L. monocytogenes* causes, in children born from infected mothers, granulomatosis infantiseptica [11], meningitis and hydrocephalus [12]. Most cases of listeriosis are of food origin; however, contact with infected ruminants, considered the most susceptible species and the main reservoir of this pathogen [13], is a remarkable risk of infection.

A high incidence of first- and second-trimester spontaneous abortions among pregnant women with active brucellosis has been reported by Khan et al. [14]. Brucella species have been isolated from fetal or placental tissues in rare cases; however, it is believed that brucellosis causes fewer spontaneous reproductive disorders in humans than it does in animals, probably because of the absence of erythritol in the human placenta and fetus [15]. 

The World Health Organization (WHO) highlighted that human leptospirosis during pregnancy may lead to abortion, fetal death, stillbirth or congenital leptospirosis, depending on the period of pregnancy, although only a few cases have been reported [16]. The results obtained by some investigations showed that rural areas, where the highest prevalences of *Leptospira* infection in animals are detected, are at the highest risk of leptospirosis [17].

Kantsø et al. [18] found a relevant level of IgG linked to *Campylobacter*, *Salmonella* and *Yersinia* in pregnant women with occupational exposure to animals, concluding that there is a real risk of adverse pregnancy outcomes due to these pathogens, even though not frequently. 

In view of the importance of this issue, rapid and accurate laboratory diagnosis is pivotal to control reproductive failure outbreaks in ruminants. Serology can be useful to monitor a flock or herd, but not to formulate an individual diagnosis when abortion and other genital problems occur. In fact, some females can shed bacteria prior to seroconversion, whereas others may be seronegative even though they are shedding the pathogens [19]. Moreover, when vaccination is carried out, serological tests are not able to differentiate vaccinated from naturally infected animals. Finally, false-positive reactions are possible, as for the diagnosis of brucellosis, where cross-reactions between *Brucella* spp. and Yersinia spp. often occur [20].

Direct antigen and/or DNA detection methods are the currently preferred methods of reaching an etiological diagnosis, and ideally these results are confirmed by the demonstration of corresponding macroscopic and/or histopathological lesions in the fetus and/or the placenta [2,21].

In conclusion, zoonotic abortive bacteria circulate among domestic ruminants worldwide, even though with different prevalences, thus the risk of their transmission to humans is remarkable. Careful prophylaxis, and prompt and accurate diagnosis in flocks and herds, are at the core of good productivity and from a One Health perspective. Accurate anamnesis in pregnant women from farming areas can be helpful to obtain a correct diagnosis in the case of genital disorders. Furthermore, veterinarians of herds affected by zoonotic abortive agents must advise pregnant women to avoid contact with the animals. 
